# Feasibility of bioabsorbable polyglycolic acid sheet and fibrin glue therapy for ulcer sealing after gastric endoscopic submucosal dissection

**DOI:** 10.20407/fmj.2025-006

**Published:** 2025-11-05

**Authors:** Tomoyuki Shibata, Takamitsu Ishizuka, Keishi Koyama, Hyuga Yamada, Noriyuki Horiguchi, Kohei Funasaka, Ryoji Miyahara, Tomomitsu Tahara, Yoshiki Hirooka

**Affiliations:** 1 Department of Gastroenterology and Hepatology, Fujita Health University, School of Medicine, Toyoake, Aichi, Japan; 2 Internal Medicine, Rinko Hospital, Nagoya, Aichi, Japan; 3 Third Department of Internal Medicine, Kansai Medical University, Hirakata, Osaka, Japan

**Keywords:** Endoscopic submucosal dissection, Gastric cancer, Bioabsorbable polyglycolic acid sheet, Fibrin glue

## Abstract

**Objectives:**

Combined therapy using a bioabsorbable polyglycolic acid sheet (BAPGAS) and fibrin glue (FG) has been employed to prevent postoperative perforation. More recently, this therapy has been applied to ulcers that develop after endoscopic submucosal dissection (ESD) for early digestive tract tumors. This study was performed to evaluate the sealing effect of this combined therapy on ulcers that develop after ESD for early gastric tumors.

**Methods:**

This study included nine patients with early gastric cancer or adenoma who were treated with ESD. Ulcers that developed after ESD were covered with BAPGAS and sealed with FG spray. To assess ulcer bleeding and healing status, endoscopy was performed on postoperative days 1, 7, 28, and 56.

**Results:**

On days 1 and 7, clots were observed in only two patients, and no bleeding occurred in any patient. As a result, endoscopic hemostasis was not required. In one patient, scar healing was achieved by day 28. By day 56, seven of the nine patients’ ulcers had reached the scar stage, and no bleeding was observed in any patient. Changes in hemoglobin levels 1 week after ESD were not significant (from 13.1 to 12.7 g/dL). The average BAPGAS covering time was approximately 40 minutes. No correlation was found between BAPGAS covering time and ESD procedure time, dissection time, or resected area.

**Conclusions:**

Combined therapy with BAPGAS and FG is a promising new treatment for preventing bleeding from artificial ulcers following ESD and may help avoid the need for additional endoscopic hemostasis.

## Introduction

The mortality rate of patients with gastric cancer has gradually declined; however, gastric cancer remains the second most common cause of malignant neoplasms in Japan. In recent years, the number of early-stage gastric cancer cases has increased because of advances in endoscopic diagnosis.

Endoscopic submucosal dissection (ESD) is a relatively new technique developed for en bloc resection of lesions such as large early gastric cancers, and it offers a major advantage over endoscopic mucosal resection.^1,2^ ESD is performed and developed as a gastric function-preserving treatment and is a less radical option than surgical resection for early gastric cancer. Because ESD is often used to treat tumors with extensive surface areas, it is important to examine the frequency of bleeding complications following the procedure. Long-term outcomes, bleeding rates, en bloc resection rates, and treatment times have been studied previously.^3–6^ As a result of these studies, ESD has been recognized as a safe and reliable procedure.

Combined therapy using a bioabsorbable polyglycolic acid sheet (BAPGAS) (Neoveil; Gunze Co., Kyoto, Japan) and fibrin glue (FG) (Beriplast P combi-set; CSL Behring Pharma, Tokyo, Japan) has been employed in many cases to prevent postoperative perforation. As a bioabsorbable material, BAPGAS is hydrolyzed and absorbed approximately 15 weeks after surgical application. Consequently, this approach has been used for ulcers requiring short-term sealing.^7,8^ Recently, the combined therapy has gained attention in the field of endoscopy for its sealing effect on ulcers that develop following ESD for early-stage digestive tract tumors.^9^

This study was performed to evaluate the sealing effect of combined therapy using BAPGAS and FG on ulcers that developed after endoscopic treatment for early gastric cancer.

## Methods

### Patients receiving ESD treatment

This study included nine patients with early gastric cancer or adenoma (eight men, one woman; mean age, 73.0 years) who were treated with ESD at our endoscopic center.

Patients with systemic diseases, malignancies in other organs, or those who had taken nonsteroidal anti-inflammatory drugs were excluded. Written informed consent was obtained from all participants. The study protocol was approved by our University Ethical Committee (HM13-223).

Gastric cancer treatment was conducted in accordance with the expanded criteria outlined in the national cancer treatment guidelines.^10^ All lesions were diagnosed as mucosal cancer following ESD.

### ESD procedure

ESD was performed using an insulation-tipped diathermic knife (IT knife; Olympus, Tokyo, Japan) to remove tumors with a minimum lateral margin of 5 mm. A high-frequency electrosurgery device (VAIO 300D; ERBE Co. Ltd., Tübingen, Germany) was used with the following settings: argon plasma coagulation at 40 W or coagulation at 30 W for marking, endo cut effect 3 at 80 W for dissection, and endo cut effect 3 at 80 W or coagulation at 50 W for abrasion. The injection solution for local administration was prepared by mixing 2 g of glycerin (20 mL), 80 mg of 0.4% sodium hyaluronate (20 mL), and 2 mL of indigo carmine solution.

### BAPGAS and FG treatment for ulcers

**Figure 1:**
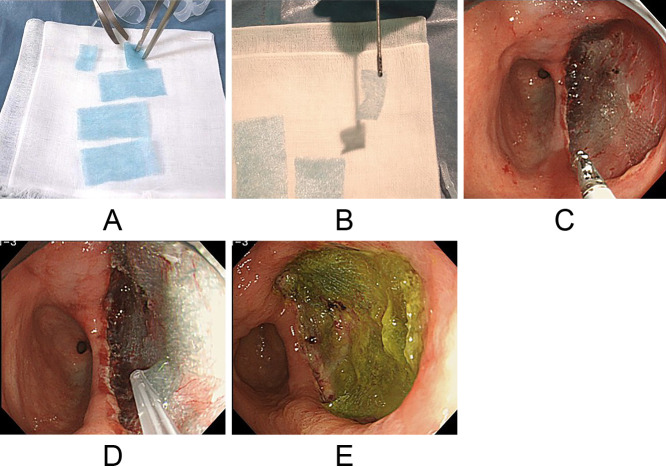
Preparation of BAPGAS and method of adhesion to the artificial ulcer. (A) BAPGAS was cut into small pieces using scissors. (B, C) A piece of the sheet was placed onto the ulcer using forceps. (D) The attached BAPGAS sheets were firmly adhered using FG spray. (E) Endoscopic image showing a successfully treated ulcer with BAPGAS on postoperative day 1.

Ulcers that developed after ESD were covered with BAPGAS and sealed using FG spray. Specifically, the BAPGAS sheet was cut into small pieces, placed onto the ulcer surface, and then firmly adhered by spraying FG (Figure 1A–D). A proton pump inhibitor, esomeprazole (AstraZeneca Co. Ltd., Tokyo, Japan), was administered for 2 weeks, and a mucosal protective agent, rebamipide (Otsuka Pharmaceutical Co. Ltd., Tokyo, Japan), was used concurrently for 8 weeks.

To evaluate bleeding and ulcer healing status, endoscopic examinations were performed on postoperative days 1, 7, 28, and 56.

### Examination of factors associated with BAPGAS therapy

Post-ESD complications, including bleeding and perforation, were assessed. Tumor locations were classified using the UML system, which divides both the lesser and greater curvatures into three equal segments: upper (U), middle (M), and lower (L), in accordance with the Japanese gastric cancer treatment guidelines.^11^ Tumor morphology was also described following these guidelines. Procedure-related data were recorded, including the total ESD time (min), endoscopic dissection time (defined as the duration of mucosal dissection only), and resected area (mm^2^). Hemoglobin (Hb) levels were measured within 1 week prior to ESD and again on postoperative days 1 and 7.

### Statistical analysis

All statistical analyses were performed using JMP version 10 (SAS Institute, Cary, NC, USA). Data are expressed as mean±standard error. The Wilcoxon signed-rank test and Spearman’s rank correlation test were used for statistical evaluations. A p-value of <0.05 was considered statistically significant.

## Results

### Patient characteristics

Patient characteristics, tumor locations, and final diagnoses are summarized in Table 1. Only one case of adenoma was identified, and no metastases were detected by pathological examination.

### Ulcer status

**Figure 2:**
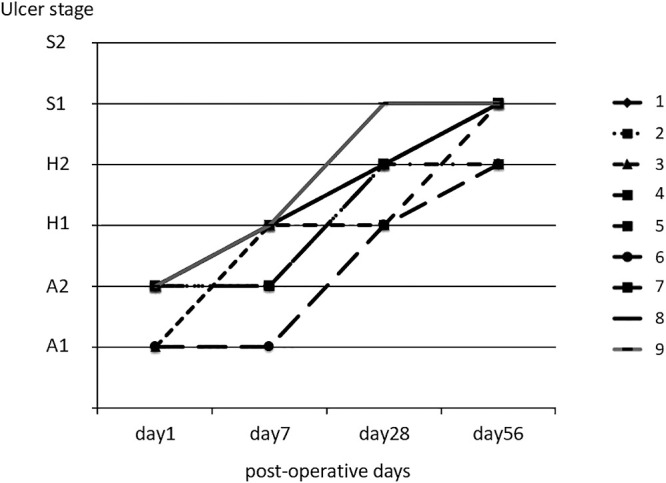
Ulcer status following ESD. By Day 56 after ESD, all ulcers had reached stage H2 or higher.

On days 1 and 7 after ESD, clots were observed on the ulcer in only two patients, and no bleeding was noted in any patient; thus, endoscopic hemostasis was not required (Figure 1E). In one patient, scar healing was achieved 28 days after ESD. By 56 days post-ESD, scar healing was observed in seven of the nine patients, and no bleeding was reported in any patient (Figure 2).

### Changes in Hb levels after ESD

**Figure 3:**
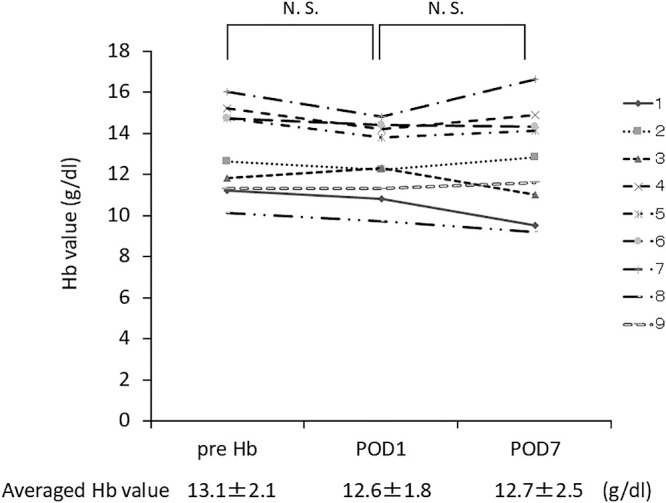
Changes in Hb levels after ESD. Hb levels showed no significant change on postoperative day 1 or 7. NS, not significant. Data analyzed using the Wilcoxon signed-rank test.

Changes in Hb levels before and after ESD were not statistically significant (from 13.1±0.7 to 12.6±0.6, p=0.0703) (Figure 3). Additionally, Hb levels 1 week after ESD (12.7±0.8) showed no change compared with those on the day 1 post-procedure (Figure 3).

### Analysis of factors associated with BAPGAS covering time

**Figure 4:**
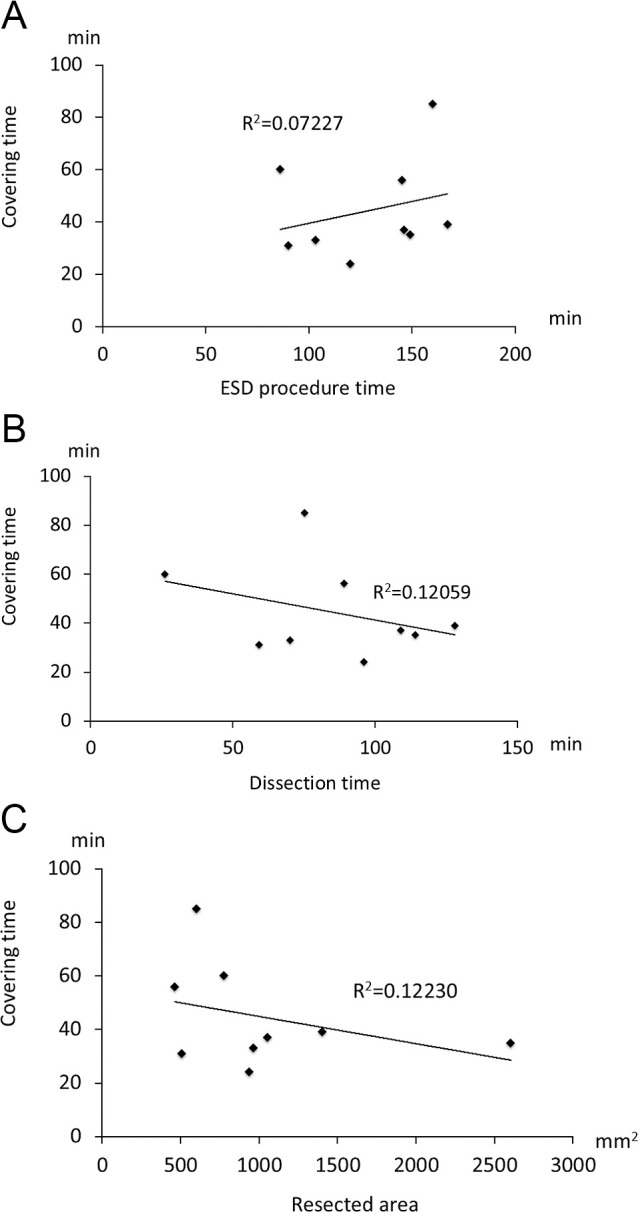
Analysis of factors correlated with BAPGAS covering time. (A–C) No significant correlations were found between BAPGAS covering time and endoscopic procedure time, endoscopic dissection time, or resected area. Data analyzed using Spearman’s rank correlation test.

The average endoscopic dissection time was 85 minutes, and the total average ESD procedure time was 130 minutes. Accordingly, the BAPGAS covering time was approximately 40 minutes (41±12 min). No factors were found to be significantly associated with the BAPGAS covering time (Figure 4A–C).

## Discussion

This is the first feasibility study to evaluate the usefulness of combined therapy with BAPGAS and FG for preventing bleeding from artificial ulcers that develop after gastric ESD. In this study, we examined endoscopic treatment time, the frequency of endoscopic hemostasis, ulcer healing speed, and Hb levels following gastric ESD.

Today, ESD is widely used in the treatment of digestive tract cancers, particularly early gastric cancer. Empirically, it is considered safer than surgery. However, postoperative adverse events can still occur, including bleeding from or perforation of the ulcer that forms after ESD. The postoperative bleeding rate is approximately 5%, while the perforation rate generally ranges from 2% to 4%.

We measured Hb as a marker of postoperative anemia. Hb levels did not show a significant decrease. Only a few reports have included Hb measurements in relation to endoscopic treatment, and detailed analyses of potential changes in Hb levels due to such procedures remain limited.^12,13^ Some studies have investigated delayed bleeding and its associated risk factors, identifying variables such as tumor size and location.^14–17^ However, findings on tumor location as a risk factor have been inconsistent. Only one study has examined the relationship between delayed bleeding and operation time,^14^ and it found no association between the two. In that study, operation time was measured in hours, whereas in our study, we used minutes. This difference in measurement units may explain the discrepancy in findings regarding the relationship between ESD procedure time and postoperative anemia.

Because BAPGAS and FG therapy is also known to help prevent delayed perforation,^8,9^ there is a possibility that these covering sheets could interfere with the prompt healing of ulcers. To evaluate this, we monitored ulcer healing speed and status after ESD using endoscopy on days 7, 28, and 56 post-procedure. We found that all ulcers had progressed to at least the second stage of healing, and the healing status was deemed clinically satisfactory. Takao et al. created ESD-induced ulcers in a porcine model and compared ulcer healing between a group treated with BAPGAS and FG and a control group.^18^ Although no significant differences in overall healing were observed, the BAPGAS with FG group demonstrated abundant neovascularization and good granulation tissue. These findings suggest that the use of BAPGAS with FG may positively influence the ulcer healing process. However, these results should be confirmed in a large-scale study.

In conclusion, combined therapy with BAPGAS and FG is effective for preventing bleeding from artificial ulcers that develop after ESD and may help shorten the overall procedure time by reducing the need for endoscopic hemostasis. Furthermore, because the ulcer healing speed was comparable to that achieved with conventional treatment, this method shows promise as a new approach for managing post-ESD artificial ulcers.

Notably, this study was conducted at a single facility with a small sample size. Therefore, multicenter randomized controlled trials are needed to further validate and confirm these findings.

## Figures and Tables

**Table 1:** Characteristics of the patients included in the study

Case	Age (years)	Sex	Location	Type	Final diagnosis
1	75	M	L, AW	0-IIa+IIc	tub2, T1a, ly0, v0
2	64	M	L, PW	0-IIa	adenoma
3	87	M	M, AW	0-I	tub2, T1a, ly0, v0
4	75	M	L, AW	0-IIa+IIc	tub1, T1a, ly0, v0
5	60	M	M, LC	0-IIa+IIc	tub1, T1a, ly0, v0
6	68	M	L, PW	0-IIa	tub1>tub2, T1a, ly0, v0
7	74	M	gastric tube	0-IIc	tub1, T1a, ly0, v0
8	79	F	M, AW	0-IIa	pap=tub1>por, T1a, ly0, v0
9	75	M	L, AW	0-IIc	tub1, T1a, ly0, v0

M, male; F, female; L, lower part; M, middle part; AW, anterior wall; PW, posterior wall; LC, lesser curvature; tub2, moderately differentiated tubular adenocarcinoma; tub1, well-differentiated tubular adenocarcinoma; pap, papillary adenocarcinoma; ly, lymphatic invasion; v, venous invasion
